# *FKBP14* kyphoscoliotic Ehlers–Danlos syndrome misdiagnosed as Larsen syndrome: a case report

**DOI:** 10.1101/mcs.a006281

**Published:** 2023-06

**Authors:** Amy Wiegand, Rama Kastury, Arpita Neogi, Arya Mani, Allen Bale, Allison Cox

**Affiliations:** 1 Smilow Cancer Genetics and Prevention Program, Yale New Haven Health, New Haven, Connecticut 06510, USA;; 2 Department of Pediatric and Adolescent Gynecology, National Institutes of Health, Bethesda, Maryland 20892, USA;; 3 Department of Cardiovascular Medicine, Yale School of Medicine, New Haven, Connecticut 06510, USA;; 4 Department of Genetics, Yale School of Medicine, New Haven, Connecticut 06510, USA;; 5 Department of Pathology and Laboratory Medicine at University of Rochester Medical Center, Rochester, New York 14642, USA

**Keywords:** common carotid artery dissection, congenital kyphoscoliosis, generalized neonatal hypotonia

## Abstract

Hereditary connective tissue disorders have overlapping phenotypes, particularly in regard to musculoskeletal features. This contributes to the challenge of phenotype-based clinical diagnoses. However, some hereditary connective tissue disorders have distinct cardiovascular manifestations that require early intervention and specific management. Molecular testing has increased the ability to categorize and diagnose distinct hereditary connective tissue disorders. A 42-yr-old female with a clinical diagnosis of Larsen syndrome from birth presented for genetic testing based on her recent diagnosis of premenopausal breast cancer. She had a past medical history of multiple carotid dissections. As she never had confirmatory molecular genetic testing for Larsen syndrome, whole-exome sequencing was utilized to assess both hereditary cancer predisposition syndromes and connective tissue disorders. A homozygous pathogenic variant in the *FKBP14* gene was identified associated with *FKBP14* kyphoscoliotic Ehlers–Danlos syndrome. We recommend that patients with a clinical diagnosis of Larsen syndrome undergo broad-based molecular sequencing for multiple hereditary connective tissue disorders. Molecular diagnosis is particularly crucial for all individuals who have a history of significant vascular events in the setting of a clinical diagnosis only. Early diagnosis of a hereditary connective tissue disorder with vascular features allows for screening and subsequent prevention of cardiovascular events.

## INTRODUCTION

There are more than 200 connective tissue disorders with known genetic etiologies, and many have overlapping phenotypes. Musculoskeletal features in particular are quite similar across hereditary connective tissue disorders, such as Ehlers–Danlos syndrome (EDS), Marfan syndrome, Larsen syndrome, and some types of skeletal dysplasias (Murphy-Ryan et al. 2010). Variable expressivity among these conditions contributes to the challenge of phenotype-based clinical diagnosis.

Importantly, establishing a diagnosis has significant implications for risk reduction, prevention, management, and follow-up, specifically in regard to cardiovascular manifestations. For example, Loeys–Dietz syndrome and Marfan syndrome are hereditary connective tissue disorders that benefit from beta-blocker therapy, antagonists of TGF-β signaling, and early surgical intervention to reduce the risk of vascular, specifically aortic, dilation, and dissection (Ritelli and Colombi 2020). The risk for cardiovascular events or abnormalities varies among connective tissue/skeletal dysplasia syndromes and the type and aggressiveness of therapy are dictated by the specific underlying genetic defect. With the advent of molecular testing, classifying and diagnosing hereditary connective tissue disorders is increasingly definitive (Murphy-Ryan et al. 2010).

Here, we demonstrate the value of broad DNA-based testing and multidisciplinary care to establish a genetic etiology in a female patient who carried an incorrect clinical diagnosis of Larsen syndrome from birth.

## RESULTS

### Clinical Presentation and Family History

**Figure 1.**
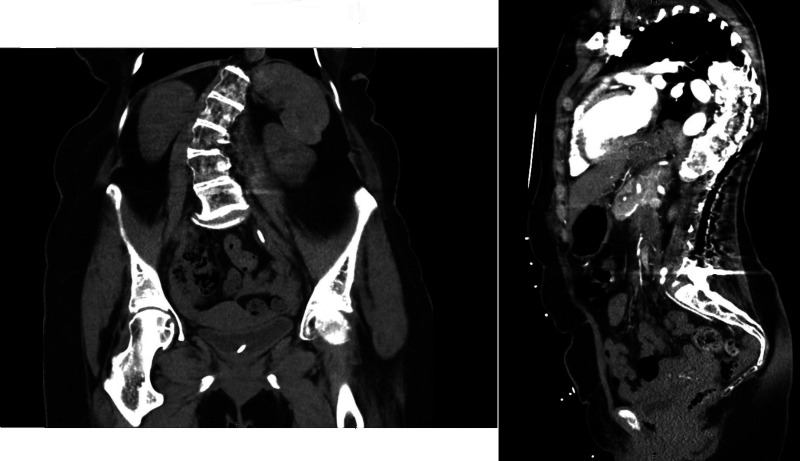
Computed tomography (CT) of the abdomen and pelvis with and without i.v. contrast of patient showing severe S-shaped scoliosis (*left*) and CT angiography (CTA) of the chest showing kyphoscoliosis.

**Figure 2.**
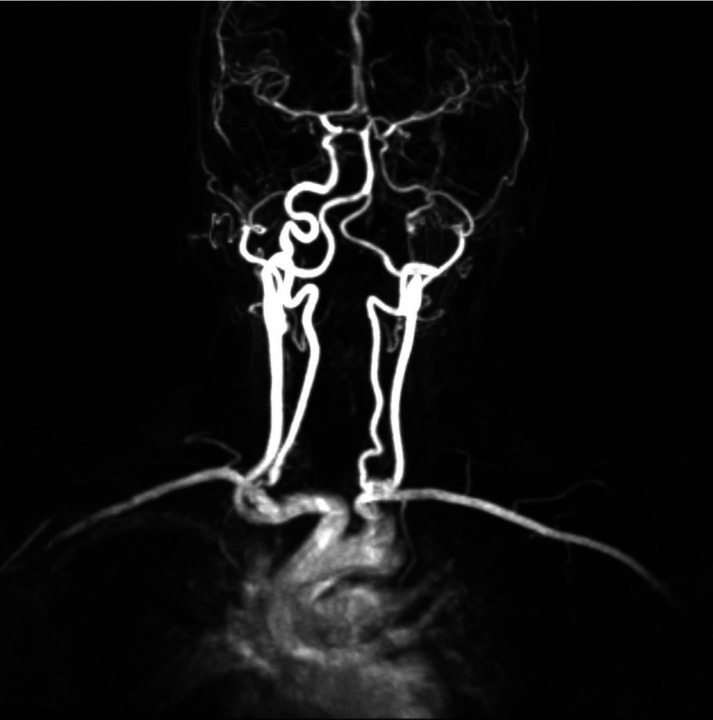
Magnetic resonance angiography (MRA) of the neck with and without i.v. contrast of patient at age 40 showing severely tortuous internal carotid arteries

The proband is a 42-yr-old female who presented to our Cancer Genetics and Prevention Program because of her recent diagnosis of invasive ductal carcinoma of the right breast. Review of her past medical history revealed that she was diagnosed clinically with Larsen syndrome at birth because of congenital hypotonia and kyphosis (Fig. 1) and had a spinal fusion with spinal rods placed at age 10. She never had confirmatory molecular testing for Larsen syndrome. The patient had her first carotid dissection at age 27 and second dissection in the contralateral carotid artery at age 32, 14 days postpartum. Additionally, she had a left hemispheric ischemic stroke at age 27 and a right middle cerebral artery stroke at age 40, which was thought to be secondary to either stenosis or thrombus associated with her right internal carotid artery dissection (Fig. 2). Since her stroke at age 40, she has been on long-term warfarin treatment. Other medical history included bleeding complications from previous dental surgery.

The patient had no family history of carotid dissection, scoliosis, or congenital hypotonia and no other family history of Larsen syndrome–associated features. She was born to nonconsanguineous parents and is of Polish, Irish, Puerto Rican, English, and non-Ashkenazi Jewish ancestry.

### Genomic Analyses

Given her personal history of early-onset bilateral carotid dissections, which is atypical for classic Larsen syndrome, and the lack of confirmatory molecular testing for Larsen syndrome, there was concern for a different hereditary connective tissue disorder explaining her cardiovascular events. Therefore, the option of an exome sequence analysis to assess both hereditary cancer predisposition syndromes and connective tissue disorders was pursued. High-throughput sequencing focused on clinically significant variants in genes associated with hereditary cancer, connective tissue disorders including *FLNB* (which is associated with Larsen syndrome)*,* and carotid dissection (Supplemental File 1).

No clinically significant variants were identified in genes associated with hereditary cancer or in the *FLNB* gene. A homozygous pathogenic variant in the *FKBP14* gene termed c.362dupC (p.Glu122fs) was identified (ClinVar ID VCV00279809.45) (Table 1). Biallelic *FKBP14* pathogenic variants are associated with *FKBP14* kyphoscoliotic Ehlers–Danlos syndrome. Although this disorder is rare, the c.362dupC variant identified in the patient has been reported multiple times and is one of the most frequently reported pathogenic variants in *FKBP14* (Baumann et al. 2012; Giunta et al. 2018; Ruiz-Botero et al. 2019).

**Table 1.** Details of pathogenic variant identified on exome sequencing analysis

Gene	Chromosome	HGVS DNA reference	HGVS protein reference	Variant type	Predicted effect	dbSNP/dbVar ID	Genotype	ClinVar ID
*FKBP14*	7	c.326dupC	p.Glu122fs	Duplication	Frameshift	rs542489955	Homozygous	VCV00279809.45

Sources: U.S. National Library of Medicine 2022; National Center for Biotechnology Information 2023.

The patient was subsequently referred for a cardiovascular genetics consultation. Physical exam revealed hypertelorism, low-set ears, high-arched palate, and pectus excavatum. Physical exam also revealed hypermobile wrists and lumbar joints. She has no history of hearing loss. Beta-blocker therapy and a computed tomography (CT) of the chest/abdomen to rule out aortic dilation were recommended. She began taking bisoprolol daily and continued anticoagulation treatment based on her past history of strokes through her breast cancer treatment. Anticoagulation treatment has been well-tolerated, and she has no active bleeding. Since her diagnosis of *FKBP14* kyphoscoliotic Ehlers–Danlos syndrome, she has had no additional vascular episodes or complications. As of the date of this report, she has not yet had a dedicated CT of the chest/abdomen.

The patient provided both written and oral consent for research and publication for this case report. This study was determined to be exempt from full IRB review under category 45 CFR 46.104(d)(4).

## DISCUSSION

Here, we describe a case of *FKBP14* kyphoscoliotic Ehlers–Danlos syndrome (*FKBP14*-kEDS) that was misdiagnosed as Larsen syndrome for 42 years until germline testing was performed during an assessment for hereditary cancer predisposition.

*FKBP14*-kEDS is a rare autosomal recessive variant of EDS, although the exact prevalence is unknown. Its hallmark features include severe congenital hypotonia, progressive kyphoscoliosis, joint hypermobility, and skin hyperextensibility. Some individuals have congenital sensorineural hearing loss (Brady et al. 2017); however, most individuals develop hearing loss in childhood. Hearing loss is most commonly sensorineural; however, conductive and mixed hearing losses have also been reported (Baumann et al. 2012; Giunta et al. 2018). A consistent facial dysmorphology has not been reported; however, some individuals may have micrognathia, hypotelorism, and a long narrow face. Although hypotonia is severe at birth and may result in delayed motor development, the majority of individuals are able to walk by age 3. Cardiovascular events have been described in a few individuals, including a celiac artery pseudoaneurysm rupture in an adult patient and an unspecified aortic rupture in a child (Brady et al. 2017). In a recent series, a patient was described with an internal carotid artery dissection at age 50, similar to the cardiovascular event seen in the current case (Giunta et al. 2018). Although *FKBP14*-kEDS is considered to be a nonvascular type of EDS, one study determined that out of the all “nonvascular” types of EDS, arterial dissections are most frequently reported in *FKBP14*-kEDS (D'hondt et al. 2018).

**Figure 3.**
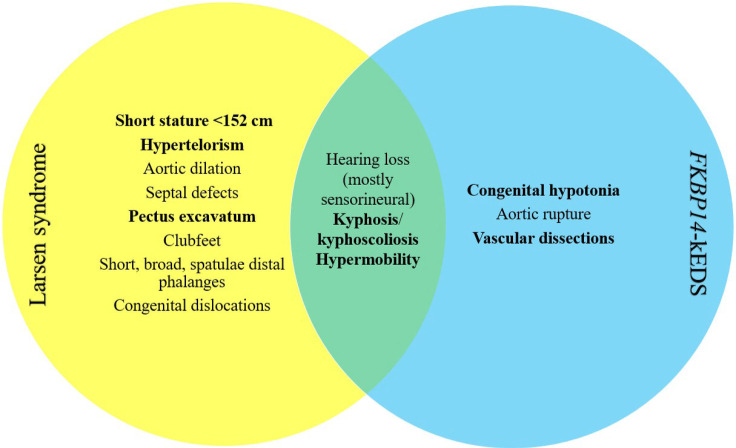
Overlapping major features of Larsen syndrome and *FKBP14*-kEDS. Features in bold represent clinical features seen in our case.

Larsen syndrome is caused by heterozygous pathogenic variants in *FLNB* and is inherited in an autosomal dominant manner. Discovered in 1950, it is probably more familiar to clinicians than kyphoscoliotic Ehlers–Danlos syndrome, and for this reason patients with overlapping features of these two disorders may be more likely to be given a clinical diagnosis of Larsen syndrome (Larsen et al. 1950). In contrast to *FKBP14*-kEDS, Larsen syndrome is a hereditary connective tissue disorder characterized by congenital dislocations, specifically of the hip, knee, and elbows, with equinovarus or equinovalgus foot deformities. Other hallmark features include spatulate fingers, most often involving the thumb, and distinct facies (hypertelorism, prominence of the forehead, depressed nasal bridge, and flattened midface) (Bicknell et al. 2006). The common spinal findings of Larsen syndrome are very similar to the hallmark kyphoscoliosis seen in *FKBP14*-kEDS. Although cardiac abnormalities have been described in cases of Larsen syndrome, specifically aortic dilation and congenital septal defects, they are not a common feature (Kiel et al. 1983). Figure 3 shows the overlapping features of Larsen syndrome and *FKBP14*-kEDS with our case's phenotype.

Although the advent of molecular testing has made diagnosis of hereditary connective tissue disorders more accurate, the phenotypic overlap among many genetically distinct conditions warrants large, expanded panel testing. In one study, focusing on Marfan syndrome's phenotypic overlap with other connective tissue disorders, 36 different clinical diagnoses that shared at least one feature in common with Marfan syndrome were identified through PubMed or OMIM literature search (Murphy-Ryan et al. 2010). These conditions involved more than 40 different genes or gene loci. Therefore, a broad differential diagnosis should be applied to any patient presenting with features of a connective tissue disorder.

Riise et al. (2018) described a 19-yr-old male with a clinical diagnosis of Larsen syndrome at birth. At age 10, he was hospitalized for a ruptured ascending aortic aneurysm, and genetic testing identified a pathogenic variant in both *TGFBR2* and *COL2A1*. These findings resulted in a dual diagnosis of Loeys–Dietz syndrome Type 2 and Stickler syndrome. Our case describes a similar scenario in which a patient was diagnosed with Larsen syndrome clinically at birth but was found to have a different hereditary connective tissue disorder through clinical genetic testing. Earlier identification of the pathogenic *FKBP14* variant and consequent diagnosis of the associated kEDS would have allowed for preventative screening and high-risk pregnancy management, and the subsequent prevention of cardiovascular events. Specifically, preventative screening may have detected an aneurysm in this patient, and an accurate molecular diagnosis circa 2012 when *FKBP14*-kEDS was discovered may have allowed for timely initiation of beta-blocker therapy, thereby reducing her risk of sequelae.

We recommend that patients with a clinical diagnosis of Larsen syndrome consider undergoing broad-based sequencing for multiple hereditary connective tissue disorders. This recommendation is extended for the clinical management of all individuals with a diagnosis or suspicion of any connective tissue disorder who have a history of significant vascular events including aortic dissection or other major vessel rupture. In general, when a phenotype sparks strong suspicion for a genetic disorder, regular reanalysis or retesting to identify variants in newly identified genes is recommended. This justification is supported by systematic studies of new clinically relevant findings identified via reanalysis of prior genetic data (Liu et al. 2019; Schmitz-Abe et al. 2019; Ji et al. 2021).

## ADDITIONAL INFORMATION

### Data Deposition and Access

Genomic data was not deposited into any public databases as consent from the patient was not obtained. The identified pathogenic variant described in this paper was deposited into the ClinVar database (https://www.ncbi.nlm.nih.gov/clinvar/) and can be found under accession number SCV003924401.

### Ethics Statement

This study was determined to be exempt from full IRB review under category 45 CFR 46.104(d)(4). The study participant provided written consent for this report to be published.

### Data Availability

The data that support the findings of this study are available from the corresponding author, A.W., upon reasonable request.

### Author Contributions

A.W., A.C., and R.K. designed and initiated the study. Drafting the manuscript and figures: A.W., A.C., R.K., and A.N., A.C., and A.B. collected data and analyzed the genome sequencing. A.W., A.N., and A.M. provided clinical care to the patient and the clinical descriptions in the manuscript. A.B. and A.M. made the final revisions. All authors read and approved the final manuscript.

### Competing Interest Statement

The authors have declared no competing interest.

## Supplementary Material

Supplemental Material
