# Supplementary material for: FKBP14 kyphoscoliotic Ehlers–Danlos syndrome misdiagnosed as Larsen syndrome: a case report
Source: Cold Spring Harb Mol Case Stud. 2023 Jun;9(3):a006281. doi: 10.1101/mcs.a006281 (PMC10393184; doi:10.1101/mcs.a006281)
Supplement: Supplemental Material [file supp_9_3_a006281__DC1.html]

Supplemental Material 

# *FKBP14* kyphoscoliotic Ehlers–Danlos syndrome misdiagnosed as Larsen syndrome: a case report

## Supplemental Material

- Supplemental\_Material.docx
