# Supplementary material for: FKBP14 kyphoscoliotic Ehlers–Danlos syndrome misdiagnosed as Larsen syndrome: a case report
Source: Cold Spring Harb Mol Case Stud. 2023 Jun;9(3):a006281. doi: 10.1101/mcs.a006281 (PMC10393184; doi:10.1101/mcs.a006281)
Supplement: Supplemental Material [file supp_mcs.a006281_Supplemental_Material.docx]

**Supplementary File**

Whole Exome Sequencing Methods

Genomic DNA was isolated from blood using the Maxwell 16 Blood DNA Purification Kit (Promega). Whole exome sequencing (WES) was performed at the Yale Center for Genome Analysis (West Haven, CT). Following amplification with IDT UDI primers, enrichment of the exome was performed by IDT xGen Human Exome V1 reagents. All libraries were sequenced using an Illumina NovaSeq-6000 with 101 bp paired-end reads. FASTQ files were aligned to the hg19/GRCh37 reference genome and variants were called, recalibrated, and filtered using GATK best practices (Van der Auwera et al., 2013). Variants were annotated with allele frequency, variant effects, OMIM and ClinVar database information, and numerous in silico attributes using ANNOVAR (Wang et al., 2010), and alignments were visualized using Integrative Genomics Viewer (Thorvaldsdóttir et al., 2013). Variants identified in the proband were filtered using a minor allele frequency cut-off of 2.5% and including only variants within or near exons.

Variants in genes associated with either Connective Tissue Disorders or Hereditary Cancer were queried for possible disease-causing variants. The homozygous *FKBP14* pathogenic variant (NM_017946, c.362dupC, p.Glu122fs, ClinVar ID: 279809) was identified as the cause of the proband’s medical history of kyphosis, carotid artery dissections, and other presentations consistent with a connective tissue disorder.

**Table S1. Whole Exome Sequencing Quality Metrics**

| **Exome Quality Metrics** | |
| --- | --- |
| Read Length | 99 |
| Number of reads (million) | 331.9 |
| Number of bases (Gb) | 32.9 |
| Mean coverage | 194 |
| PCR duplicates | 24.73% |
| Multiply mapped | 4.38% |
| Unmapped | 0.07% |
| 10x target base coverage | 99.0% |
| 30x target base coverage | 98.7% |
| 50x target base coverage | 98.1% |
| 100x target base coverage | 89.2% |
|  | |
| **Variant Quality Metrics** | |
| Alternate allele depth | 124 |
| Total depth | 125 |
| Mapping Quality | 60.08 |
| Genotype Quality Score | 99 |
| Filter | PASS |

References

Van der Auwera, G. A., Carneiro, M., Hartl, C., Poplin, R., del Angel, G., Levy-Moonshine, A., Jordan, T., Shakir, K., Roazen, D., Thibault, J., Banks, E., Garimella, K., Altshuler, D., Gabriel, S., & DePristo, M. (2013). From FastQ data to high-confidence variant calls: The genome analysis toolkit best practices pipeline. Current Protocols in Bioinformatics, 43, 11.10.1–11.10.33.

Wang, K., Li, M., & Hakonarson, H. (2010). ANNOVAR: Functional annotation of genetic variants from next-generation sequencing data. Nucleic Acids Research, 38, e164.

Thorvaldsdóttir, H., Robinson, J. T., & Mesirov, J. P. (2013). Integrative Genomics Viewer (IGV): High-performance genomics data visualization and exploration. Briefings in Bioinformatics, 14, 178–192.
